# Key steps and suggestions for a promising approach to a critical care mentoring program

**DOI:** 10.1186/s44158-023-00116-4

**Published:** 2023-08-29

**Authors:** Silvia De Rosa, Denise Battaglini, Victoria Bennett, Emilio Rodriguez-Ruiz, Ahmed Mohamed Sabri Zaher, Laura Galarza, Stefan J. Schaller, on behalf of the NEXT Committee of the ESICM

**Affiliations:** 1 Centre for Medical Sciences (CISMed), University of Trento, 38122 Trento, Italy; 2 Anesthesia and Intensive Care, Santa Chiara Regional Hospital, APSS Trento, Trento, Italy; 3 Department of Anesthesia and Intensive Care, IRCCS Ospedale Policlinico San Martino, Genoa, Italy; 4 Department of Intensive Care Medicine, St George’s University Hospital NHS Foundation Trust, London, UK; 5 Intensive Care Medicine Department, University Clinic Hospital of Santiago de Compostela (CHUS), Galician Public Health System (SERGAS), Santiago de Compostela, Spain; 6 Adult Intensive Care Unit, Oxford University Hospitals NHS Foundation Trust, Level 1, John Radcliffe Hospital, Headley Way, Oxford, OX3 9DU UK; 7 Department of Intensive Care, Hospital General Universitari de Castelló, Castelló de La Plana, Spain; 8 Department of Anesthesiology and Surgical Intensive Care, Charité — Universitätsmedizin, Berlin Institute of Health, Freie Universität Berlin, Humboldt-Universität Zu Berlin, Berlin, Germany

**Keywords:** Career development, Critical care, Matching, Medical education, Mentee, Mentor, Mentoring

## Abstract

In critical care medicine, where there is a demanding career with a problematic work-life balance, mentoring is an important support tool to grow professionally, creating a network of support throughout the career. The mentoring process consists of evidence-based steps to guide critical care mentors and mentees and pair them with each other according to the correct selection and matching of participants.

In order to focus on the active role of a young intensivist selected as a mentee at any level and to support their success in a mentoring relationship, the NEXT Committee of the European Society of Intensive Care Medicine (ESICM) developed 2012 a mentoring program.

The critical steps of the mentoring program start from establishing a policy and program objectives, passing through the selection of participants, and matching with mentors up to the definition of the personal development plan supported by checklists, worksheets, and evaluation forms. The present manuscript provides key steps and tips for a good, essential based on our experience in the ESICM NEXT-Mentoring Program so that they guide for future mentoring programs conducted by other scientific societies. In addition, we discuss common challenges and how to avoid them.

**Supplementary Information:**

The online version contains supplementary material available at 10.1186/s44158-023-00116-4.

## Introduction

Mentoring is an essential process and an exchange relationship to advance the careers and careers of the mentor and the mentee [1]. Furthermore, mentoring is an important strategy to help recruit, retain, and develop research projects and critical care expertise. In medicine, mentoring has been recognized as a core component of training and career advancement in academic medicine [2–5]. Berk et al. and Buddeberg-Fischer et al. [6, 7] specified three key elements and objectives of mentoring relationships: (1) direct interaction, (2) long-lasting, and (3) integrated approach including emotional and psychological support as well as direct assistance with career and professional development. Previous studies have shown that successful mentoring is based on matching the mentee and mentor according to personal and attitudinal similarities [8–11]. Additionally, a mentoring program consists also in a collaborative opportunity to grow professionally, thus creating a network of support. Indeed, both the mentor and the mentee can benefit from having someone to talk to about the constant daily pressures, about research projects, and about how to achieve the right work-life balance. Mentoring is also one of the innovative short-term solutions that have been in place in many healthcare institutions to tackle human resource-related challenges in low- and middle-income countries (LMICs) [12].

In order to focus on the active role of a young intensivist selected as a mentee at any level and to support their success inside a mentoring relationship, the NEXT Committee of the European Society of Intensive Care Medicine (ESICM) developed in 2012 a mentoring program. In 2020, we implemented the “NEXT-Mentoring program” to facilitate mentoring relationships between students and faculty mentors, who participated in a formal mentoring program that fosters early introduction to research and promotes academic careers. In the present manuscript, we will discuss key steps and tips for a good approach, essential based on our experience in the ESICM NEXT-Mentoring program, so that they are a guide for future mentoring programs conducted by other scientific societies. In addition, we discuss common challenges and how to avoid them.

### Mentoring program

Critical care is a multi-professional community; teamwork is paramount in our daily practice. The diversity of skill sets, experience, and knowledge of the critical care team requires collaboration in the delivery of care, research, and professional development. In this setting, staff satisfaction, retention, and effective leadership can increase staff morale, decrease medical turnover rates, and improve patient care quality. Unfortunately, mentoring programs in critical care are built more for the nursing staff and less for the medical staff [13], particularly in a low-income country [14].

Nowadays, mentoring has evolved from the traditional dyad of a mentor and mentee to a more extensive network of collaborators based on cornerstone characteristics. The definition and elaboration of an innovative mentoring program in a critical area, as suggested by Everett Rogers, must be based on the following fundamental principles:

1. Relative advantage—The perception of change among the group
2. Compatibility—The importance of values and past experiences of innovation
3. Simplicity—The ease of understanding the proposed change
4. Trialability—The degree to which innovation can be tested
5. Observability—More discernible results contribute to how comprehensively the innovation will be adopted (see Rogers 1).

In support of these principles, peering can be a significant tool for sharing skills and ideas among all groups: mentees share with mentors, and mentors share with other mentees and mentors [15]. Particularly, skills shared and acquired from a networking relationship are also crucial in transcultural considerations [16, 17]. On the other hand, there are many human factor challenges in critical care, and the following strategies can improve collaboration: (1) recognition of skills and clinical expertise, (2) providing positive reinforcement for excellence in multidisciplinary networking, (3) identifying mentoring and precepting experiences, (4) participating at local, regional, and national conferences, (5) understand working conditions and identify different personality needs, (6) recognition of critical care-related stress impact and investigate ways of handling them, and (7) the climate should be one of reflection, openness, and communication [18, 19].

### Practical steps for mentoring process

**Fig. 1:**
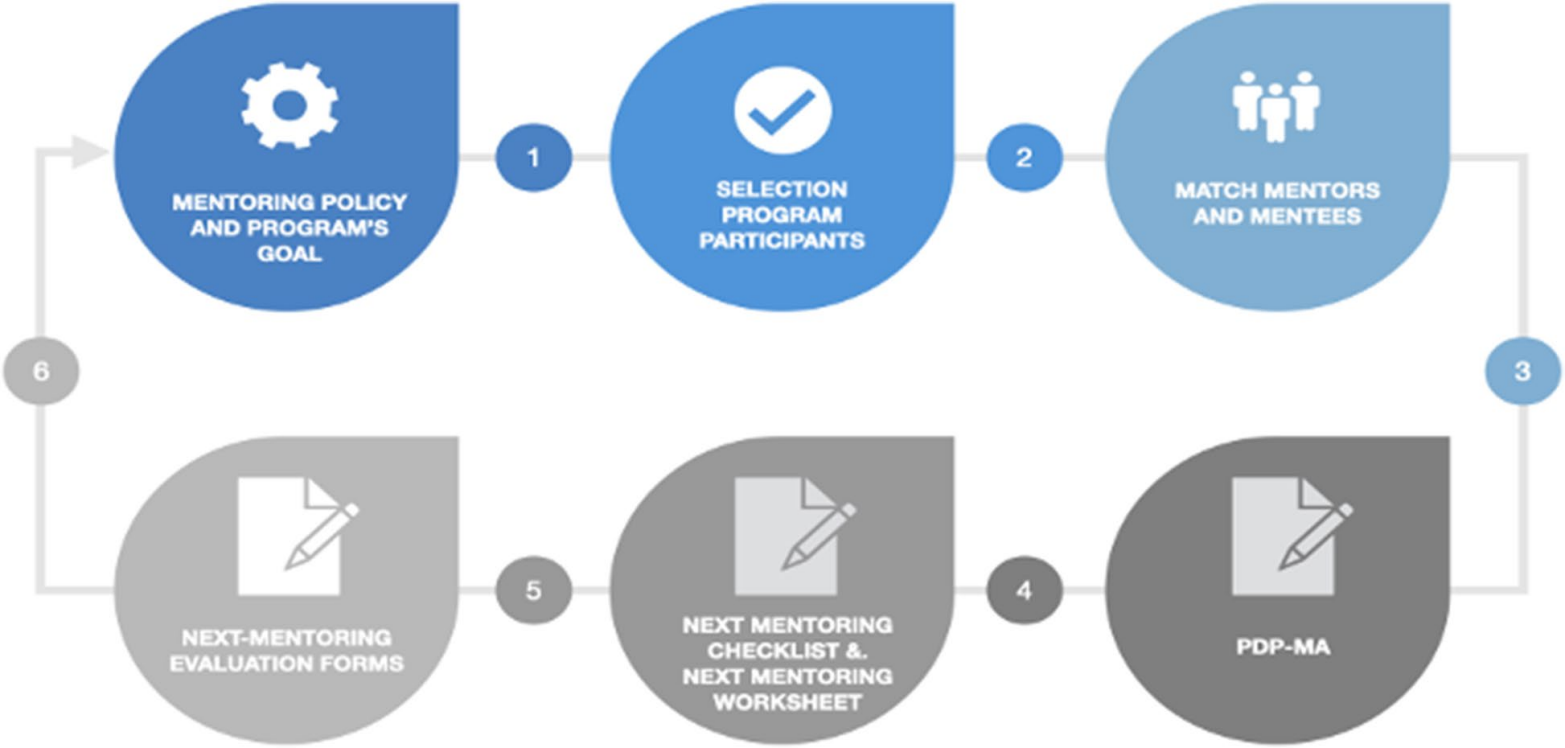
The six-step mentoring process. PDP-MA, personal development plan and mentoring agreement

The mentoring process is an evidence-based process used to help guide critical care mentors and mentees and pair them with each other according to a correct selection and matching of participants. Mentors and mentees are at the heart of the mentoring program. They do not enjoy meeting when they are not compatible with each other. The role of the ESICM NEXT in the mentoring program is to advertise the program, elicit participation, judiciously select mentees, contact mentors for each new mentee in the program, support the flow of the program, review program feedback and outcome metrics regularly to improve the program organization, and develop education workshops. The steps to build a mentoring program are described and explained below (Fig. 1), partly inspired by the mentoring program performed at the Center for Simulation, Advanced Education and Innovation at Children’s Hospital of Philadelphia (CHOP).

### Establish a mentoring policy and program goals

The ESICM considers mentoring a key component of training, research experience, and career development. Therefore, ESICM’s policy is to support, endorse, and promote mentoring.

Through the leadership and guidance of a robust research program, the ESICM NEXT-Mentoring Program aims to develop professional and successful individuals, guiding them on their career paths. Consequently, this mentoring program strives to establish, design, and facilitate positive, enduring, and mutually beneficial mentoring team relationships that allow mentees to plan, learn, collaborate, and grow in their clinical and research careers. The ESICM NEXT-Mentoring Program focuses on managing a productive academic career and nurturing and cultivating junior mentees to become the next generation of research leaders, encouraging professional development, and establishing effective and independent researchers. Ultimately, this program allows mentees to broaden their professional networking within the scientific community.

**Fig. 2:**
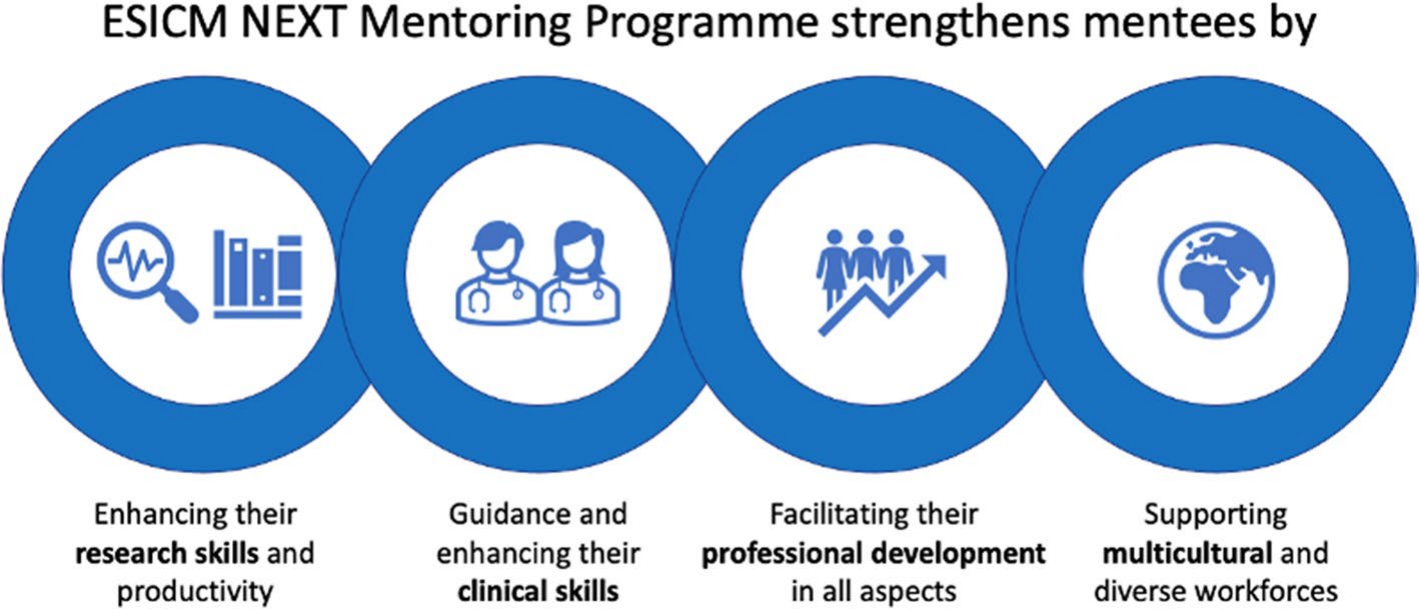
Mentoring policy and objectives

The specific objectives of the ESICM NEXT-Mentoring Program (Fig. 2) are to enhance and support a candidate’s research career and strengthen mentees by the following:

- Enhancing their research skills and productivity
- Guidance and enhancing their clinical skills
- Facilitating their professional development in all aspects
- Supporting multicultural and diverse workforces

### Selection program participants

The fulcrum of mentoring is represented by the mentees who are the program’s recipients and without devaluing a two-way relationship that can be of equal value for both mentees and mentors. For this reason, it is strongly recommended to select the mentees first, allowing the coordinator to assess the suitability of mentors in relation to the profiles and needs of the mentees. High levels of motivation and career needs are on the list of mentee selection criteria; However, this program is also for young talents eager to expand their quantitative research methods and practices. For this reason, an ESICM NEXT mentee should possess basic research and clinical knowledge, problem-solving and innovation skills, the ability to work independently, value research integrity and collaborative research, and good communication skills and interpersonal skills. During the assessment of applicants, it is also considered age, gender, and regional location, ensuring inclusivity.

Each section of ESICM provides a list of mentor candidates which are selected based on their experience and contribution to society. These potential mentors are then contacted individually via email by the program coordinator, who explains the structure and responsibilities of the mentor but also of the mentee within the program. The coordinator guarantees the presence as a support to the future developing relationship. Both components of this report have responsibilities to consider, which will be further explored later.

### Match mentors and mentees

One of the most challenging aspects of a program that can influence productivity is performing a good match [20]. Participants will bring various competencies, backgrounds, learning styles, and needs [21]. Matching is a challenging process to conduct and should be appropriate. Participants give different contributions, from other skills to different learning styles and needs. In addition, barriers that can affect mentoring have been identified, such as in residency in surgical surgery, including time constraints and lack of female mentors [22]. The pairing is based on the expected skills and areas of interest between mentor and mentee. However, if a mentoring program aims to improve careers, mentors must be in a higher position than mentees. To avoid mentees or mentors dropping out, it is necessary to carry out the coupling adequately by following the *essential key points*.

First of all, the coordinator of a mentoring program should know *the goals of individual mentees* (i.e., research improvement, clinical skills improvement, increase in talent retention) [23]. With a clear purpose, the coordinator can make significant assumptions in the matching process, guessing which criteria makes sense to match instead of mapping it to the individual’s organizational goals in the program. This can cause an incorrect match and dissatisfaction in the couple with a perception of loss of time between the people involved [24]. Identifying the primary goal of the program mentoring program will guide the coordinator through the rest of the matching process. A survey could support this case, as reported in the literature on surgical training [20].

Secondly, *profiles and criteria* for matching mentors and mentees should be created in order to match them on the right skill traits and fill out rich profiles [25].

Thirdly, it will be necessary to determine *the type of matching for mentors and mentees* [25]. To this end, several criteria could be used to determine which correspondence will work best, including the number of participants or the requirements to participate as a mentor or mentee. There are four types of matching:

1. *Self-matching* — That consists in which mentees find their own mentors career based on interests and/or skills [26]; Shollen et al. found that mentee-initiated matching resulted in increased career satisfaction [27, 28].
2. *Admin matching* — In which the mentoring program coordinator creates matches on behalf of the participants [25]
3. *Bulk matching* — That allows to match a large pool of program participants at the same time (more than 200 participants) [29]
4. *Hybrid matching* — This method of matching mentors and mentees requires you to use the questions asked for manual matching. From this, you will create a pool of mentors for the mentee to select from.

The NEXT Mentoring is based on *admin matching mentors and mentees that is a manual matchmaking* process performed based on the selected number of participants [10] for a high-quality program.

In case of more than 10 mentees, software available can help pair mentors and mentees using advanced algorithms that automatically suggest the best match by analyzing the data in each participant’s profile in order to find the best mentoring matches. Online platforms facilitate the creation of mentoring networks that provide mentees with specific, accessible, and timely support [30].

Fourthly, *providing a guide* for the couple is necessary [31]. Unfortunately, this is often overlooked. There will be powerful and productive couples and others that need support. To this end, the coordinator must try to support the couple in every way, even by scheduling remote or face-to-face meetings. Unfortunately, help is needed in some couples to ensure mentor and mentee progression.

### Personal development plan and mentoring agreement

At the first meeting, mentees should have a plan, predefined goals, and need [32]. The personal development plan and mentoring agreement (PDP-MA) is a contract or agreement between the mentor and mentee to clearly define and outline a road map of the mentee’s development goals to create a personally tailored action plan to develop specific competencies, improve actual performance, and gain new responsibilities. The main objective of the PDP-MA is to provide a program that helps facilitate mentee development, identifying what the mentee wants to learn and the skills, knowledge, and attributes they want to work on to achieve the defined goals. Moreover, it should help identify the mentee’s leadership competencies and strengths that might be useful in achieving the goals.

The PDP-MA of the NEXT-Mentoring Program is organized as a form to be filled by the mentor and mentee with the following information: a section dedicated to identifying the personal goals of the mentee, the reason for applying for the NEXT-Mentoring Program, hopes for the career, and a list of other goals discussed with the mentor. Additionally, the document presents a section dedicated to the areas of interest of the mentee, including expectations regarding the main objectives of the NEXT-Mentoring Program: (1) teaching activity, (2) research activity, (3) personal development, (4) networking, and (5) other (if any) to be by a mentor.

Each of these possible objectives requires information on (1) which are the goals, (2) uses of resources need of collaborators and time required to achieve goals, and (3) barriers to achieving new goals. In the PDP-MA, the frequency of meetings between mentor and mentee is defined, as well as the expectations for this mentoring relationship, while describing the expected responsibilities of both mentor and mentee. The complete form is available as Supplementary Material Table 1.

### NEXT-Mentoring checklist and NEXT-Mentoring worksheet

The mentee and mentor have specific responsibilities to ensure that the relationship is effective, and that objectives are met. Both mentee and mentor should follow semi-structured guidance that is created for the mentor to teach the mentee in particular skills, as well as to facilitate the growth of the mind by sharing resources and networks, challenging the mentee to overcome the comfort zone, and creating a safe learning environment, and for the mentee to facilitate their knowledge and progresses in lives and career. A checklist of the NEXT-Mentoring Program has been developed to clarify the responsibilities and goals of the mentor and mentee and is provided in Table 1.

**Table 1:** Mentee and mentor’s checklist

	**Mentee’s tasks**	**Mentor’s tasks**	**Documents to be utilized**
**Prior to your first next-mentoring meeting**	Ask yourself — what are my goals? How can a mentor assist me in meeting these goals? What are my competency levels as a teacher, researcher, clinician, administrator, and in the community?	Be sure that your mentee knows how to contact you. Request contact information from your mentee	PDP-MA
	Take the initiative. Stay connected to your mentor. Invite your mentor to meet in face or via videoconferencing; suggest potential topics. Agree on confidentiality and no-fault termination	Stay connected with the mentee and encourage her/him	
	Update your own CV	Obtain mentee’s CV prior to the first meeting so that you already know pertinent professional information	
	Consider the skills that require additional mentoring: what skills do I need to learn or improve? What do I want to change about my work style? Which professional networks are important?	Review and discuss the specific aims that you and your mentee developed. Discuss your expectations and your needs with your mentee. Work with your mentee on goals for the relationship (meeting time, etc.). Plan to meet at least twice a year	
	Complete the PDP which provides a framework for you and your mentor to review your personal and professional career goals	Review and discuss the PDP-MA; complete and sign the mentoring agreement	
	Develop a list of specific aims on which you want to collaborate on with your mentor If among your objectives there is the need to perform a clinical and/or research training at the department or institute of your assigned mentor, consult our E-MOVE platform at the following link: https://www.esicm.org/emove/public/	Provide focused guidance on “schedules” and what events and meetings the mentee is expected to attend	
	Schedule an online meeting with the coordinator or with the employees of the Next-Mentoring program. This can be a useful moment to ask for explanations or clarifications on the program	Help your mentee develop a checklist that you both can follow	
	Agree with your mentor upon the following: • Duration and start/end dates of mentoring, possible attendance at the research institute or hospital, economic support guaranteed by the mentor, if possible • Time of first meeting, location, and agenda	Agree with your mentee upon the following: • Duration and start/end dates of mentoring, possible attendance at the research institute or hospital, economic support guaranteed by the mentor, if possible • Time of first meeting, location, and agenda	
**First mentoring meeting**	The first meeting should be at the ESICM-LIVES mentor–mentee meeting	The first meeting should be at the ESICM-LIVES mentor–mentee meeting	Mentoring worksheet Mentoring evaluation form
	Review and discuss your PDP-MA with your mentor. Discuss your short- and long-term goals and expectations and work together to develop steps to reach these goals using a timeline	Review and discuss the PDP-MA with your mentee	
	Discuss and critique the specific aims	Discuss and critique the specific aims	
	Determine frequency of meetings. This will vary based on individual needs. We suggest planning at least two meetings a year besides the meeting at LIVES mentor–mentee lunch. The extent of the interaction can vary from phone calls or Internet meetings to face-to-face meetings	Determine frequency of meetings	
	Suggest potential topics for future meetings (examples: setting and achieving goals, managing time effectively in an academic environment, balancing personal and professional life, negotiating for what you want/need, completing manuscripts)	Encourage the mentee choosing potential topics for future meetings	
**After the first meeting and throughout the mentoring**	Establish your own checklist for follow-up. Keep an ongoing portfolio of activities & works in progress. Check your timeline	Evaluate and discuss the mentee checklist and timeline	Mentoring worksheet Mentoring evaluation form Mentoring coordinator support
	Meetings with mentor should be scheduled	Meetings with mentee should be scheduled	

Table legend: *PDP* Personal development plan, *MA* Mentoring agreement

Both mentors and mentees should take notes during the meeting to monitor the progress and follow up on agreed-upon action plans (activity report). The NEXT-Mentoring Program coordinator or collaborators will contact mentees and mentors every 6 months to receive feedback and support if necessary. Both mentor and mentee can contact the program manager for any help. At the end of the second year (and subsequent years) and/or at the end of the mentoring relationship, the mentee and mentor should provide a final review to the NEXT-Mentoring Program coordinator. In any case, mentors or mentees may reassess their relationship at any time during the mentoring program and decide to continue or discontinue. If a mentor–mentee pair chooses to stop, a discontinuation evaluation form should be filled out.

### Joint responsibilities

#### Responsibilities of the mentor

The mentor should act as a facilitator, a guide, a supporter, and a supervisor by providing constructive criticism and feedback. These responsibilities are achieved by formalizing goals, objectives, and metrics of success with the mentee, meeting periodically to review progress and attainment of goals, providing feedback and encouragement on accomplishments, evaluating the mentee’s development during the program, and being assessed by the mentee, with an assessment of whether the mentor meets or exceeds expectations.

#### Responsibilities of the mentee

The first responsibility of the mentee is to contact the assigned mentor to plan the activities. Additional tasks of the mentee include taking part in the mentor–mentee lunch meeting at ESICM-LIVES for two consecutive years, initiating contact and scheduling meetings with a mentor, preparing a specific agenda for each session, including career goals, and learning to evaluate the ideas and suggestions provided by the mentor.

The goals that meet expectations will be evaluated by the mentor and assessed by the mentee using the NEXT-Mentoring worksheet during the mentoring program. All the main 4 objectives of the program (teaching, research, networking, self-development) plus others, if any, will be evaluated for accomplishment and obstacles. The mentor and mentee will plan new strategies to overcome the barriers together (Supplemental Material Table 2).

A NEXT-Mentoring evaluation form is a tool designed to evaluate our mentoring training, composed of 50-item skills (8 open and 42 ranked questions) for the mentee and 32-item skills (8 open and 24 ranked questions) for the mentor. These sheets are developed to give feedback about the relationship, attitudes, and feelings about acquired competencies. They include questions about professional development, professional career development, skill development, personal communication, role model, mentorship program quality, partnership, personal growth as mentee and mentor, and relationship. The form uses a 5-point Likert scale for ranked questions to allow the mentee and mentor to express how much they agree or disagree with a particular statement. The Likert scale provides five possible answers to a statement or question that allows respondents to indicate their positive-to-negative strength of agreement or strength of feeling regarding the question or statement, rated as follows: 1 = strongly disagree, 2 = disagree, 3 = neither agree nor disagree, 4 = agree, and 5 = strongly agree. The scale has a rank order, but the intervals between values cannot be presumed equal [33, 34]. Supplementary Material Tables 3 and 4 provide the complete NEXT-Mentoring evaluation forms.

### Challenges

The mentoring program can be very demanding and require dedication, but it has challenges. Firstly, the availability of mentors could be extremely limited by personal and work commitments that would not allow the mentor to follow the assigned mentee properly. Secondly, while on the one hand, the mentor can be excessively busy; on the other hand, very often, the mentee is afraid to express personal and career needs [35, 36]. Thirdly, some mentors enter the mentoring program to seek out a mentee like themselves, but without avoiding psychological conditioning [37]. Sometimes the disappointments are related to advice or suggestions not followed by the mentor, also probably associated with a poor adaptation to the personality [38]. Fourthly, although the NEXT-Mentoring Program is remote mentoring, enabling support for individuals who are not geographically mobile or who lack local mentors with relevant expertise not available, misunderstandings could be due to telephone and e-mail communications being overcome [39].

The possible solutions to the problems above can be, in our opinion, the following:

1. The introduction of the figure of coordinator in a mentoring program indeed allows for an accurate selection of mentors, a well-defined matching, and support if required in the mentor–mentee relationship without being invasive or insensitive.
2. The selection of mentors must be accurate and directed not only towards the extreme technical experience in specific sectors but also above all availability, enthusiasm, emotional intelligence, active integrity in mentoring, active listening, and honest feedback.
3. The mentee should be educated about the potential difficulties they may encounter while building the relationship with their mentor. Decline the relationship in the sign of reciprocity ensures that the interpersonal encounter does not remain confined to a purely utilitarian level or one of domination/overwhelm, but it should be directed and structured around the principles of respect and equality dignity [38].
4. The first face-to-face meetings or a period of attendance carried out in the mentor’s office could be helpful at the beginning of the NEXT-Mentoring Program [39, 40], in order to strengthen the relationship.

## Conclusion

Although numerous studies have considered the potential role for mentoring, there are few dedicated mentoring programs for critical care trainees and young specialists and no formalized training opportunities for those wishing to act as mentors. Mentoring has a valuable role to play in enabling mentees to achieve their extensive potential, while developing leadership and interpersonal skills, and to become also potential future mentors.

In the present manuscript, we described the planning and type of support we created for our mentoring program in ESICM NEXT, which can be a pocket guide for future mentoring programs conducted by other scientific societies. Although the mentoring program for a scientific society is an impactful strategy to develop and engage people, running an impactful program goes way beyond just matching people up. It takes effort, resources, time, and dedication. We hope that these suggested tips will likely improve the mentor and mentees’ communication and experience.

### Supplementary Information

**Additional file 1: Supplementary Material Table 1.** PDP-MA. **Supplementary Material Table 2.** NEXT-Mentoring worksheet. **Supplementary Material Table 3.** NEXT-Mentoring evaluation form mentee. **Supplementary Material Table 4.** NEXT-Mentoring evaluation form mentor.

## Data Availability

Not applicable.
