# Supplementary material for: Key steps and suggestions for a promising approach to a critical care mentoring program
Source: J Anesth Analg Crit Care. 2023 Aug 29;3:30. doi: 10.1186/s44158-023-00116-4 (PMC10464173; doi:10.1186/s44158-023-00116-4)
Supplement: Supplementary file 1 — Additional file 1: Supplementary Material Table 1. PDP-MA. Supplementary Material Table 2. NEXT-Mentoring worksheet. Supplementary Material Table 3. NEXT-Mentoring evaluation form mentee. Supplementary Material Table 4. NEXT-Mentoring evaluation form mentor. [file 44158_2023_116_MOESM1_ESM.docx]

**Supplementary Material**

**Table of contents**

Table 1 – PDP-MA [page ]

Table 2 - NEXT-Mentoring worksheet [page ]

Table 3 - NEXT-Mentoring evaluation form mentee [page ]

Table 4 - NEXT-Mentoring evaluation form mentor [page ]

**Supplementary Material – Table 1**

| ***Development Goals and Plan*** | | |
| --- | --- | --- |
| **Identify Personal Goals** | *Why did you decide to apply for the NEXT-Mentoring Program?* | |
|  | *What do you hope to accomplish in your career?* | |
|  | *List other goals discussed with your Mentor* | |
| **Areas of Interest: Definition of Effort** | **Teaching** | Student and/or resident teaching, student advising, curriculum teaching, new course development, etc. |
|  | **Research Activity** | Conducting research, presentations and publications, funding and grant support and application, copyrights and patents, editing, and peer review. |
|  | **Personal Development** | Development of activities, leadership programs, active participation in professional academic associations or societies. |
|  | **Networking** | Developing professional contacts, expanding network contacts, utilizing additional mentors in specific areas of focus. |
|  | **Others** | To be defined in concordance with Mentor |
| **Specific Goals in Focus Areas** | Complete the focus areas that specifically apply to the criteria for your mentorship and therefore will help you accomplish your personal short- and long-term goals. | |
|  | **Teaching** | Teaching goal(s) |
|  |  | *Identify resources, collaborators, and time needed to achieve goal(s)* |
|  |  | *Identify barriers to achieve new goal(s)* |
|  | ***Research and Research Related Activities*** | *Identify the focus of your research activity.* |
|  |  | *Research goal(s)* |
|  |  | *Identify resources, collaborators, and time needed to achieve goal(s)* |
|  |  | *Identify barriers to achieve new goal(s)* |
|  | ***Self-Development*** | *Self-Development goal(s)* |
|  |  | *Identify resources, collaborators, and time needed to achieve goal(s)* |
|  |  | *Identify barriers to achieve new goal(s)* |
|  | **Networking** | *Networking goal(s)* |
|  |  | *Identify resources, collaborators, and time needed to achieve goal(s)* |
|  |  | *Identify barriers to achieve new goal(s)* |
|  | **Others** | To be defined in concordance with Mentor |
| **Topics that will be addressed in mentoring sessions** | | |
| Teaching | | YES/NO |
| Research | | YES/NO |
| Self-Development | | YES/NO |
| Networking | | YES/NO |
| **Frequency of meeting with the mentor** | | |
| Monthly | | YES/NO |
| Bi-monthly | | YES/NO |
| Three-monthly | | YES/NO |
| Quarterly | | YES/NO |
| **Review, discuss, edit and check the expectations for this mentoring relationship** | | |
| Responsibility of the mentor | | Provide assessment and feedback concerning accomplishments in each topic |
|  | | Help plan “next steps” |
|  | | Advocacy |
|  | | Actively address any problems with mentorship relationship |
|  | | Other (please specify) |
| Responsibility of the mentee | | Provide goals and updates |
|  | | Actively address any problems with mentoring relationship |
|  | | Other (please specify) |

**Supplemental Material – Table 2**

| **GOALS** | **EXPECTATIONS** |
| --- | --- |
| **Goal: Research** | ⬜ Not Expected  ⬜ *Meets Expectations*  ⬜ *Below Expectations* |
| Accomplishments: | open question |
| Obstacles: | open question |
| New goal or strategy to overcome obstacles (if needed): | open question |
| **Goal: Teaching** | ⬜ Not Expected  ⬜ *Meets Expectations*  ⬜ *Below Expectations* |
| Accomplishments: | open question |
| Obstacles: | open question |
| New goal or strategy to overcome obstacles (if needed): | open question |
| **Goal: Networking** | ⬜ Not Expected  ⬜ *Meets Expectations*  ⬜ *Below Expectations* |
| Accomplishments: | open question |
| Obstacles: | open question |
| New goal or strategy to overcome obstacles (if needed): | open question |
| **Goal: Self-Development** | ⬜ Not Expected  ⬜ *Meets Expectations*  ⬜ *Below Expectations* |
| Accomplishments: | open question |
| Obstacles: | open question |
| New goal or strategy to overcome obstacles (if needed): | open question |
| **Goal: other** | ⬜ Not Expected  ⬜ *Meets Expectations*  ⬜ *Below Expectations* |
| Accomplishments: | open question |
| Obstacles: | open question |
| New goal or strategy to overcome obstacles (if needed): | open question |

**Supplementary Material – Table 3**

| **Topics** | **Rating** | **Comments** |
| --- | --- | --- |
| **Professional Development** | | |
| Encourages my inventiveness including identification of new research topics and learning of new methodologies | 1 2 3 4 5 |  |
| Helps me develop my capacity for theoretical reasoning and data interpretation | 1 2 3 4 5 |  |
| Helps me to be critical and objective concerning my own results and ideas | 1 2 3 4 5 |  |
| Helps me become increasingly independent in identifying research questions and conducting and publishing my research | 1 2 3 4 5 |  |
| Provides constructive feedback on my experimental designs | 1 2 3 4 5 |  |
| Provides thoughtful advice on my research progress and results | 1 2 3 4 5 |  |
| **Professional Career Development** | | |
| Provides counsel for important professional decisions | 1 2 3 4 5 |  |
| Provides opportunities for me to meet with visiting scientists, faculty and peers | 1 2 3 4 5 |  |
| Maintains balance between pursuing his/her own research and developing my own career | 1 2 3 4 5 |  |
| Helps me to envision a career plan | 1 2 3 4 5 |  |
| Provides guidance in development and presentation of research projects for outside review groups | 1 2 3 4 5 |  |
| Ensures that I am firmly grounded in rules regarding ethical behavior and scientific responsibility | 1 2 3 4 5 |  |
| **Skill Development** | | |
| Helps me to work effectively with other individuals | 1 2 3 4 5 |  |
| Helps me to develop good negotiating skills | 1 2 3 4 5 |  |
| Provides constructive feedback on my presentation and writing skills | 1 2 3 4 5 |  |
| Encourages me to present my work at scientific meetings | 1 2 3 4 5 |  |
| **Personal Communication** | | |
| Listens carefully to my concerns | 1 2 3 4 5 |  |
| Routinely monitors my progress and reviews proposed timelines and milestones with me | 1 2 3 4 5 |  |
| Takes into account gender, ethnic, and cultural issues in interacting with me | 1 2 3 4 5 |  |
| Does not take advantage of my time and abilities | 1 2 3 4 5 |  |
| Provides timely feedback | 1 2 3 4 5 |  |
| Helps me to clarify my responsibilities such as contributing to team effort, working diligently and responding to criticism | 1 2 3 4 5 |  |
| Is appropriately accessible to me | 1 2 3 4 5 |  |
| **Serves as Role Model** | | |
| Conveys high ethical standards and concern for research subjects | 1 2 3 4 5 |  |
| Illustrates active teamwork and collaboration | 1 2 3 4 5 |  |
| Illustrates good mentoring skills | 1 2 3 4 5 |  |
| Easy to approach and talk with | 1 2 3 4 5 |  |
| Offers advice and encouragement with respect to independent goals | 1 2 3 4 5 |  |
| Provides regular feedback and constructive criticism | 1 2 3 4 5 |  |
| Facilitates participation in professional activities outside of the institution (regional, state, national organizations) | 1 2 3 4 5 |  |
| Involves me in networking and/or invites me to informal gatherings of people from work | 1 2 3 4 5 |  |
| Advocates on my behalf within the department or division | 1 2 3 4 5 |  |
| Connects me to other senior professionals who could “fill in the gaps” in areas where you might be less skilled | 1 2 3 4 5 |  |
| Observes me in a teaching situation and provides feedback on these critical skills | 1 2 3 4 5 |  |
| Holds all to high standards | 1 2 3 4 5 |  |
| Establishes a written plan including goals to be met under the direction or guidance of my Mentor | 1 2 3 4 5 |  |
| Establishes guidelines at the beginning defining how often or when we would meet on a routine basis | 1 2 3 4 5 |  |
| Determines at the beginning of the relationship guidelines by which to evaluate the success of the relationship | 1 2 3 4 5 |  |
| **Mentorship Program Quality** | | |
| Goals of my mentorship were met | 1 2 3 4 5 |  |
| I was happy with the style of mentoring in the program. | 1 2 3 4 5 |  |
| The mentoring program met my expectations. | 1 2 3 4 5 |  |
| The mentoring program was a positive experience | 1 2 3 4 5 |  |
| **Your Partnership** | | |
| What are/were the most beneficial development activities you did/do with your Mentor? |  | |
| What is the most beneficial change you identified in yourself as a result of your mentoring? |  | |
| **Personal Growth** | | |
| As the result of having a Mentor, I’ve gained the following knowledge, skills and/or attitude change. |  | |
| Other benefits I’ve received from this mentoring relationship. |  | |
| Things I plan to do or have done more of as a result of the relationship |  | |
| **Relationship** | | |
| Ways, if any, this mentoring program could be more effective |  | |
| Recommendations I’d make to other mentor-mentee pairs |  | |
| General comments on the mentoring program |  | |

**Supplementary Material – Table 4**

| **Topics** | **Rating** | **Comments** |
| --- | --- | --- |
| **Mentoring** | | |
| My mentee and I had an understanding of our Mentoring relantionship | 1 2 3 4 5 |  |
| My mentee was motivated to assume his/her responsabilities | 1 2 3 4 5 |  |
| Our meetings (in face or via video conferencing) were puposeful and timely | 1 2 3 4 5 |  |
| My mentoring training was beneficial | 1 2 3 4 5 |  |
| I had adequate time for Mentoring | 1 2 3 4 5 |  |
| My mentee and I followed an action plan | 1 2 3 4 5 |  |
| I was supported by ESICM Next Mentoring Coordinator in the mentoring process | 1 2 3 4 5 |  |
| **Mentee’s Professional Career Development** | | |
| Mentoring was a rewarding experience | 1 2 3 4 5 |  |
| I was able to meet the expectation of my mentee | 1 2 3 4 5 |  |
| My Mentoring directly affected my Mentee’s advancement or retention | 1 2 3 4 5 |  |
| **Mentee’s Skill Development** | | |
| My mentee work effectively with other individuals | 1 2 3 4 5 |  |
| My mentee developed good negotiating skills | 1 2 3 4 5 |  |
| I provide constructive feedback on presentation and writing skills to my mentee | 1 2 3 4 5 |  |
| My mentee presented research project at scientific meetings | 1 2 3 4 5 |  |
| **Personal Communication** | | |
| Listens carefully to my suggestions | 1 2 3 4 5 |  |
| I routinely monitor the progress and reviews proposed timelines and milestones with me | 1 2 3 4 5 |  |
| Does not take advantage of my time and abilities | 1 2 3 4 5 |  |
| Provides timely feedback | 1 2 3 4 5 |  |
| Mentee had cleared his/her responsibilities such as contributing to team effort, working diligently and responding to criticism | 1 2 3 4 5 |  |
| Mentee was appropriately accessible to me | 1 2 3 4 5 |  |
| **Mentorship Program Quality** | | |
| Goals of my mentorship were met | 1 2 3 4 5 |  |
| I was happy with the style of mentoring in the program. | 1 2 3 4 5 |  |
| The mentoring program met my expectations. | 1 2 3 4 5 |  |
| The mentoring program was a positive experience | 1 2 3 4 5 |  |
| **Your Partnership** | | |
| What are/were two of the most beneficial development activities you did/do with your Mentee? |  | |
| What is the most beneficial change you identified in yourself as a result of your mentoring? |  | |
| **Personal Growth as a Mentor** | | |
| As the result of having a Mentee, I’ve gained the following knowledge, skills and/or attitude change. |  | |
| Other benefits I’ve received from this mentoring relationship. |  | |
| Things I plan to do or have done more of as a result of the relationship |  | |
| **Relationship** | | |
| Ways, if any, this mentoring program could be more effective |  | |
| Recommendations I’d make to other mentor-mentee pairs |  | |
| General comments on the mentoring program |  | |
